# “Like a broom tied together”: A qualitative exploration of social cohesion and its role in community capacity strengthening to support integrated health in Nigeria

**DOI:** 10.1371/journal.pgph.0002508

**Published:** 2023-10-24

**Authors:** Adetayo Adetunji, Martha Silva, Nrupa Jani Tulsiani, Mayokun Adediran

**Affiliations:** 1 Population Council, Utako, Abuja, Federal Capital Territory, Nigeria; 2 Department of International Health and Sustainable Development, Tulane University School of Public Health and Tropical Medicine, New Orleans, LA, United States of America; 3 Population Council, Washington, DC, United States of America; University of Colorado Denver - Anschutz Medical Campus: University of Colorado - Anschutz Medical Campus, UNITED STATES

## Abstract

Social cohesion, broadly understood as the degree of connectedness, solidarity, and trust across various community groups and between individuals, is critical for community capacity. This paper examines social cohesion and its role in community capacity strengthening for sustaining integrated health gains in Nigeria. This study took place in the context of a mid-course qualitative evaluation of a Community Capacity Strengthening approach that focuses on engaging Ward Development Committees (WDC) to increase community agency, coordinate and support the ward-level health ecosystem and ensure sustained community-level activities supporting behaviour change for improved health outcomes. This qualitative study was conducted in four selected wards per state in Bauchi and Sokoto states, targeting WDC members, Village Development Committee members, Community Volunteers, local government officials, traditional leaders, and Community Capacity Strengthening project staff. Thematic content analysis findings show that recognition and legitimacy were operationalized through the election of members into committees which in turn gave them a sense of identity and credibility. At the community level, WDCs leveraged the influence of social networks in the community to achieve their goal. Trust was also identified as a prerequisite to the acceptance and accomplishment of social and behaviour change programming. At the institutional level, our findings revealed strong conflict management skills and high collective efficacy of committee members for programme implementation. This study found high cohesion among committee members, promoting a sense of belonging and agency, and facilitating social and behavior change activities for improved health outcomes. However, we found clear limits to the extent to which high social cohesion can contribute to community capacity to sustain health implementation and improvements. While cohesive community organizations present a good opportunity for health programmes, there is a need for more investment of resources to address funding, logistics, and service delivery limitations.

## Introduction

While numerous definitions of social cohesion have been proposed in the literature, there is no single comprehensive definition due to different interpretations of the idea of social cohesion by institutions, policymakers, and scholars [1–3]. Nonetheless, social cohesion is broadly understood as the degree of social connectedness and solidarity between various community groups, involving the amount of trust and connection between individuals and across the community [4–7]. Likewise, although most scholars acknowledge that social cohesion is a multidimensional concept, they disagree on the precise form of these dimensions [8]. Three core dimensions of social cohesion that the majority of social cohesion scholars agree on include social relations, sense of belonging, and orientation towards the common good [9]. Fonseca, Lukosch & Brazier (2019) put forth three levels of social cohesion: the individual, community and institutions [10].

Social cohesion is particularly vital for community capacity which is the individual and aggregate strengths of members to overcome barriers and find or cultivate opportunities to improve the overall well-being of a given community as well as that of individual community members. This is in addition to collective efficacy, self-belief, conflict resolution, and leadership style that is competent [11]. Community capacity also refers to the ability to take action to address the community’s needs including the ability to gather the resources, and the social and political support necessary to solve challenges and improve health and wellbeing [12]. Another definition emphasises local initiatives that may or may not be integrated into community organizations, focusing on specific health or social issues [13]. Community capacity, strongly linked to community development, is widely recognized as crucial for programme sustainability [14]. Goodman et al. (1998) described 10 domains of community capacity: leadership, citizen participation, skills, networks, resources, sense of community, community power, understanding community history, values, and critical reflection [15]. Community capacity strengthening is viewed as an underutilized resource and can be viewed as both a means and an end for community-based health promotion efforts [16]. Community capacity strengthening empowers individuals to strategically plan, evaluate, and visually represent outcomes as they address their circumstances [17]. Also, community capacity strengthening has been linked to several benefits, including better target population reach, efficient resource utilization, higher local competence and commitment to health action and change, and increased community responsiveness to emerging health challenges [18–21]. While community capacity programmes in health strive to strengthen community engagement and mobilization [22], it is also a critical requirement for community ownership and has a long-term influence on public health [23–26].

Studies implemented in lower- or middle-income countries have consistently highlighted the significance of enhancing community capacity to foster healthcare-seeking behaviours [27–29]. There is a paucity of information regarding the role of social cohesion and its implications for community capacity strengthening in general, especially in Nigeria. For example, In Abimbola et al.’s 2016 study on community health committees in Nigeria, they identified the importance of capacity building, support, and policy-backed legitimacy. They also suggested that social cohesion, through multiple shared platforms, can enable connections beyond immediate networks, leading to improved service uptake at a lower cost for committee members. However, the study lacks sufficient evidence demonstrating how social cohesion specifically enhances community capacity [27]. In contrast, studies that have measured social cohesion in Nigeria have not done so in the context of community capacity strengthening [30, 31]. The limited evidence regarding the role of social cohesion can be attributed to the absence of a consensus on its understanding and application. This lack of agreement has made it challenging to compare studies that examine this concept [32]. Using Fonseca and colleagues’ framework [10], this paper explores three domains of community-level social cohesion in Nigeria and the extent to which these domains highlight social cohesion as its contributes to community capacity. In addition, this paper delves into the resultant threats to the sustainability of the collective action to address family planning, maternal, neonatal, child health and nutrition (MNCH+N), malaria and social outcomes such as increased community agency.

## Social cohesion framework

This study’s findings were analysed using an adapted version of Fonseca and colleagues’ social cohesion framework, which emphasizes the characteristics that play a significant role in facilitating social cohesion and explains how it may be cultivated from different perspectives. The framework consists of three separate yet interconnected levels: individual, community, and institutional, which are further divided into factors.

The individual level is divided into three factors: 1) self-motivation; 2) perceptions, norms, and values; and 3) participation and performance. The self-motivation factor is related to the reasons that motivate an individual to be in a group. It also includes topics on intimate face-to-face communication, recognition, and legitimacy. Perceptions, norms, and values refer to the individual’s perspective on the group they are a part of and their own set of beliefs. The degree of ’like-dislike’ and sense of community are two ways that this is demonstrated. The final component on this level is participation and performance, which relate to a person’s motivation to act and assume responsibility in a group. This component is influenced by initiative, individual participation, task competence, and individual behaviour.

The community level is divided into three factors: 1) the social environment; 2) relationships and ties; and 3) process performance and goal attainment. The social environment element is connected to a group’s social climate and could be associated with shared norms and values, formal/informal control of the group, friendship networks, pressures for conformity and caring for group norms, or civic society. The second factor of relationships and ties is related to social capital, trust, reciprocal loyalty and solidarity, moral support, or the value of incentives in the group. The third factor, process performance and goal attainment is concerned with the group’s performance and its common objectives, which are therefore related to common goals and moral behaviours/norms.

The institutional level involves three factors: 1) conflict management and decision-making; 2) human rights; and 3) the environment (structures, norms, and values). Conflict management and decision-making are seen as the governance of formal institutions in society and can be linked to social disorder or conflict, as well as the reduction of inequities and exclusion. The human rights factor considers the agency, access to basic needs, and freedom of the person while in a group/society. Finally, the environment (structures, norms, and values) factor refers to the formal institutions and individuals in society who are accountable for their upkeep.

**Fig 1:**
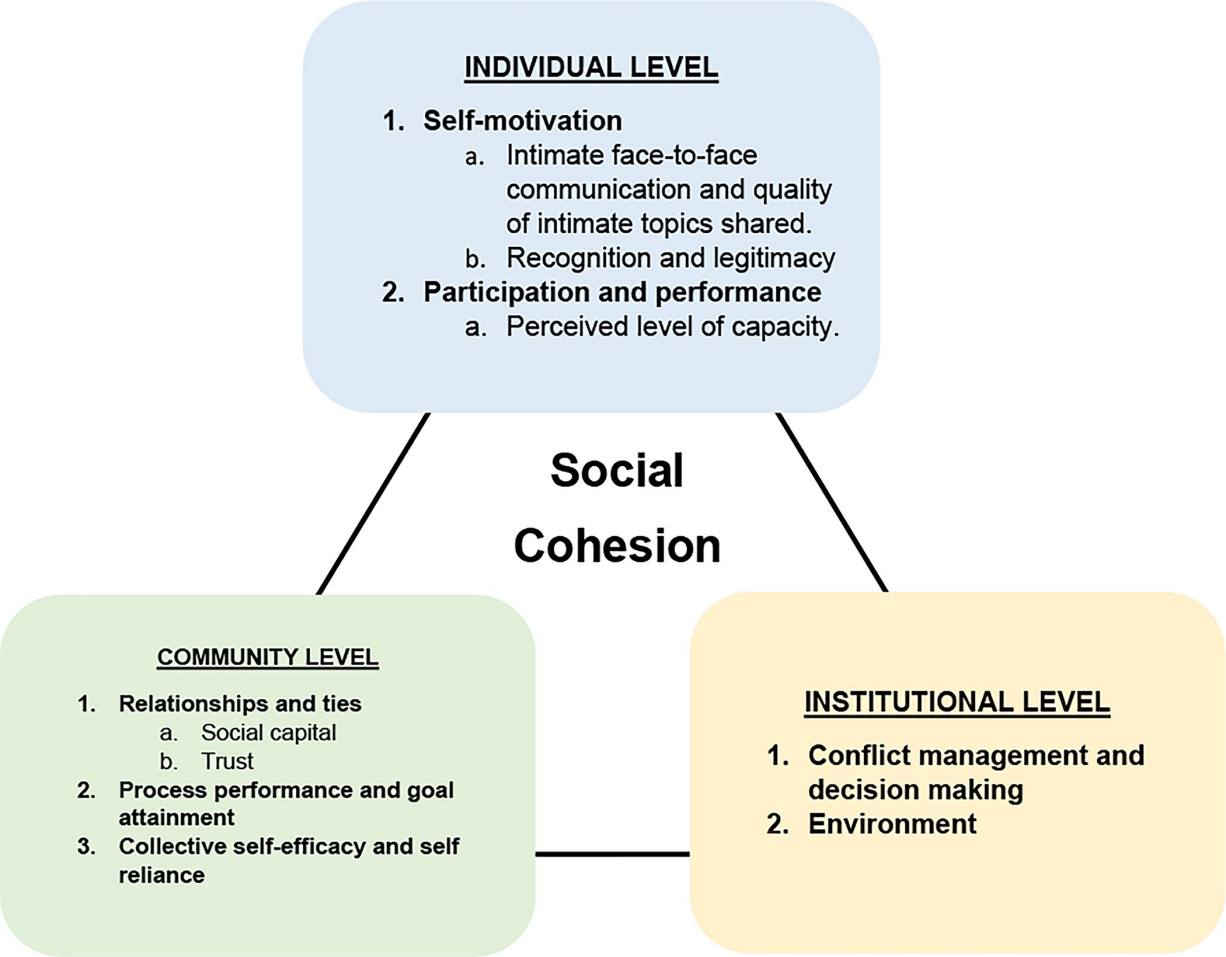
Adapted social cohesion framework.

The social cohesion framework was used to organize the study findings by addressing the three levels and corresponding factors as shown in Fig 1.

### Study context

Community health committees are community-based structures that can enable communities to participate in the governance of their primary health care [33]. Ward development committees (WDCs) and village development committees (VDCs) exemplify these committees as significant means of encouraging local leadership, credibility, engagement, and governance. National policy in Nigeria highlights the involvement of WDCs together with health workers in the co-management of primary health care facilities and service delivery [34]. Nonetheless, these committees necessitate ongoing capacity building and related support, including that delivered through social and behaviour change_(SBC) programming [27]. For example, a qualitative study that examined capacity building for WDCs to promote health in Nigeria showed that following capacity-building activities such as training, mentoring, and supportive supervision, there was increased participation and commitment by the WDCs that translated to increased support for health facilities to provide quality health services in the community [28, 35]. Community-based structures can enable communities to participate in the governance of primary health care [33].

According to the national minimum standards for primary health care in Nigeria, WDCs have a diverse set of responsibilities such as strategizing for healthcare, monitoring the implementation of pertinent work plans, mobilizing resources for community initiatives, and overseeing the activities of village health workers, all of which lead to improved health-related behaviours and increased demand for healthcare [36]. WDCs provide a platform to facilitate community participation and ownership of community engagement activities for primary health care such as community mobilization; MNCH+N [37, 38]; reproductive health; and health promotion [39]. Earlier studies have recognized the obstacles encountered by WDCs, which hinder their ability to effectively carry out their designated responsibilities. These challenges include limited access to transportation, tools, and insufficient space for meetings [39].

This study took place in the context of a mid-course qualitative evaluation of Breakthrough ACTION/Nigeria’s Community Capacity Strengthening activity that focuses on engaging community leaders and structures—namely WDCs—to increase community agency, coordinate and support the health ecosystem in general and to ensure sustained community-level activities supporting SBC for improved health outcomes [40]. The Community Capacity Strengthening intervention included an approach in which WDC members across selected wards were trained and provided technical support to support and guide monthly micro-planning meetings, monitoring and evaluation and promote data use for decision-making. The support provided allowed committee members to surmount challenges hindering the utilization of certain MNCH+N services. In this context, this paper examines community-level social cohesion and its role in community capacity strengthening in Nigeria. Using Fonseca and colleagues’ social cohesion framework [10], this paper explores three domains of community-level social cohesion in Nigeria and its implications for community capacity strengthening and sustainability of community organization members’ engagement in collective action to address family planning, MNCH+N, malaria and social outcomes such as increased community agency.

## Methods

### Study design

The study utilized a qualitative design based on in-depth interviews (IDIs), and key informant interviews (KIIs). The study was conducted in eight wards, four in Bauchi (Dankade, Ningi East, Badara, Kwagal) and four in Sokoto (Achida, Tunga, Durbawa, Gandu) in 2021. The wards were selected using maximum variation purposive sampling technique based on internal Community Capacity Strengthening implementation assessments that revealed skewed and ultimately low variability in the performance of wards at the time of selection. Consequently, the sampling technique was used to accommodate this range of project implementation performance.

### Study population

IDIs were conducted among WDC members, VDC members, Community Volunteers (CVs), local government area (LGA) officials and traditional leaders to gather insights on the potential effects of the Community Capacity Strengthening approach on the community SBC activities (see Table 1). KIIs were conducted among Breakthrough ACTION/Nigeria project staff to garner information on the implementation of community SBC activities and early transition toward community ownership. Across all population groups, information was sought on elements of social cohesion such as collective self-efficacy, conflict management, perceptions of group norms and level of capacity, and trust. It is important to mention that WDCs and VDCs are both part of the same ward health system, and according to existing primary health care guidelines, VDC representatives are meant to be included in the WDCs [41]. Additionally, Community Volunteers (CVs) are also incorporated into this ecosystem, as part of the Breakthrough ACTION SBC intervention’s objectives.

**Table 1:** Description of study activities per state.

Target Audience	Bauchi	Sokoto	Total
WDC members (IDIs)	6	8	14
VDC members (IDIs)	8	7	15
CVs (IDIs)	5	5	10
Traditional leaders (IDIs)	1	1	2
LGA officials (IDIs)	3	5	8
Breakthrough ACTION staff (KII)	5	5	10

### Instruments

The interview guides were developed and adapted based on previous research [11]. Before the commencement of data collection, study tools were pilot tested in similar wards and among similar populations. The pilot testing activity offered us the chance to identify and refine ambiguous questions, assess the suitability of the guides, determine interview length, test interview flow, and identify potential response bias or sensitive questions. The guides were drafted in English, and all were translated into Hausa. These guides played a crucial role in garnering valuable insights on community cohesion, further encompassing aspects such as competent leadership; equitable information access; community self-efficacy; gender equality; experiences related to supportive oversight; perceived factors that influence capacity strengthening and sustainability.

### Data collection

Data collection took place over two weeks, specifically from 26th July to 6th August 2021, with fieldwork conducted concurrently in the two study states. Based on the defined inclusion criteria of the study, participants were recruited with the aid of local government health educators and community mobilizers who were conversant with the terrain and understood the fundamentals of the Breakthrough ACTION/Nigeria project. Additionally, trained field assistants constantly liaised with the mobilizers to ensure that the right participants were selected for fieldwork. Following the written informed consent process, interviews were conducted face-to-face and lasted approximately an hour. The data collection followed the study’s security protocols and COVID-19 risk reduction strategies.

Research assistants engaged in reflexivity and bias awareness, reflecting on their own biases, assumptions, and preconceptions during the interviews and transcription process. Peer debriefing and team discussions took place after each interview session, allowing for immediate perspectives to be shared, addressing any challenges, and ensuring appropriate framing of questions.

### Data management and analysis

Audio recordings of all IDIs were conducted with participants’ consent. These recordings were later translated and transcribed upon the completion of data collection. by research assistants that were fluent in the local language. The transcription process involved multiple levels of review. An initial draft was created, followed by a proofreading stage where a separate researcher compared the text with the audio file, checking for errors, omissions, or inconsistencies. Finally, a thorough review of the transcript was conducted to ensure accuracy, clarity, and adherence to quality standards. NVivo 12 software was utilised for data management. Randomly selected transcripts were reviewed by six researchers to gain familiarity with the data during a review session that spanned a week. The themes revealed during the data collection phase underwent further exploration, with the research staff also identifying additional emerging themes. A codebook was created to systematize the codes that were drafted to explore the diverse themes associated with the study objectives. A team consisting of four researchers worked together to code the dataset. Regular team meetings were held to facilitate discussions and address any coding disagreements, particularly regarding the suitability, code names, and descriptions. The resolved differences were utilized to update the codebook and previously coded transcripts, ensuring consistent use of codes across the entire dataset.

We used thematic content analysis, an approach employed for interpreting textual data that involves the systematic identification of themes or patterns in text data [42]. Subsequently, emergent themes were improved using a constant comparative method. This involved comparing the identified themes to determine if similar notions surfaced within and across study sites and populations [43]. The study’s analytical rigor was greatly improved by the use of data triangulation across a variety of participant categories, which entails incorporating multiple data sources to develop a thorough understanding of the phenomenon being studied, as well as analytic triangulation, which involves the input of multiple analysts to explore different perspectives on the data [44].

Ethical approvals for the study were obtained from the Tulane University Human Research Protection Office Institutional Review Board (Approval Number 2021–210), the National Health Research Ethics Committee (Approval Number NHREC/01/01/2007-05/02/2021), and the Sokoto (Approval Number SKHREC/040/2021) and Bauchi (Approval Number NREC/03/11/19B/2021/17) State health ethical review boards.

## Results

The study’s findings are presented in accordance with the social cohesion framework’s individual, community, and institutional levels. At the different levels, findings are further organised by factors.

### Individual level factors affecting social cohesion

The individual-level factors explored in this study were self-motivation, participation and performance. In this study, self-motivation comprised face-to-face communication and the quality of topics shared as well as recognition and legitimacy of individuals in the group. Furthermore, the participation and performance factors were defined by perceived level of capacity.

#### Self-motivation

Face-to-face communication and the quality of topics shared were explored to understand individual self-motivation. Under the Community Capacity Strengthening approach, community organization members leverage regular meetings to discuss ideas on a variety of community health issues. These meetings are often held monthly and quarterly, according to participants. The quarterly meetings in particular are expanded sessions that include more stakeholders, such as the LGA Director of Health. Furthermore, emergency meetings could be organised as a result of an urgent event that necessitates action. These sessions enable members of community organizations to be on the same page regarding their activities, track progress, and resolve difficulties. These meetings result in a good level of motivation for community committee members and community volunteers, who can then plan and design ways to improve their performance.

“*Yes*, *I participated in all of these review meetings*, *where we discuss how to harmonize our activities*. *We discuss our achievements and issues encountered while doing our work*, *and we come up with solutions on how to do it better… Sometimes*, *when something happens without the month coming to an end*, *you will see an emergency gathering*, *informing people to come because something has happened in the ward*…*”*

• **Bauchi, Female CV**

“*If not for the meetings sincerely the activities we are tasked with would not even be possible…*. *The meeting itself makes it possible for us to get acquainted with each other and again we get more enlightened and understand the need for cooperation*.

• **Bauchi, Male VDC member**

*Recognition and legitimacy*. For WDC member leadership, recognition is attained through WDC elections. WDC members are elected following a meeting to assess who is interested in and would perform well in this capacity. According to participants, gender, education, speaking/persuasive abilities, community reputation of supporting others (e.g., recommending women to antenatal care), language ability, relatability, past donations (education/wealth/time) provided to benefit the community, and trustworthiness are among the criteria. WDC members in particular highlighted education as a characteristic that enhances the legitimacy and functionality of WDC members.

Gender-based recruitment, especially women’s participation, is intended to ensure WDC membership is fair/equitable. However, women’s participation is mostly determined by the availability of candidates and is hindered by social norms and practices that limit women’s participation outside of household duties.

“*Women are viewed with respect and are seen as role models because they speak to their women on health issues*. *But some men may not permit their wives to do this kind of work*.*”*

• **Sokoto, Male VDC Member**

Participants who were WDC and VDC members thought their membership as part of the community organization was rewarding. Perceived rewards include knowledge gained while performing tasks. A male WDC member in Bauchi expressed the following viewpoint.

“*I enjoy being a part of WDC because I now know so many things that I did not know before*, *yes like the things that affect the people in my ward*. *My being in WDC*, *I see that I have the authority to help them in this area*, *whereas in the past I can only watch them with my eyes I would not know what to do*.*”*

• **Bauchi, Male WDC Member**

WDC and VDC members also benefitted from increased respect and recognition from government representatives and community members alike, as a result of the critical roles they play in the community. For instance, an LGA official explained that *“WDCs help a lot of pregnant women in this community by providing transportation means to the health facility*… *they also have the capacity to help the people who do not have the financial means of helping themselves to get better services*.*”*

“*Sincerely I have gotten a rewarding experience based on the sensitization we have been doing*. *The rewards I have gotten are in praises*, *you will find out that all calibre of people giving you all manner of praise*. *They come to respect you*, *visit you at home to appreciate you and commend you for what you have been doing*. *This is a good reward*.*”*

• **Bauchi, Female VDC Member**

The voluntary nature of WDC membership was also a notable element of what brought legitimacy to their role. The financial commitment of committee chairpersons provides an example for others to follow in terms of their potential financial contribution to sustain the work and may influence others in the community beyond committee members.

“*The part that gives me joy is when I talk the people listen and get a positive result because they did exactly as they are advised*, *they believe*, *they could see my efforts knowing I am not being paid for the job that is voluntarily that is why*.*”*

• **Sokoto, Bauchi WDC Member**

Community organization members also shared the importance of having the zeal and commitment necessary to contribute to group activities. This was reflected in the opinion of a female LGA official in Sokoto state who stated that committee members, as volunteers, need to *“have the zeal and take ownership”* of their activities, which has been strengthened through the Community Capacity Building intervention.

#### Participation and performance

According to the guiding framework, the perceived level of ability relates to how strongly individuals within the committees think they are capable of participating in achieving their objectives. There was a popular notion among participants that the members of community organizations such as the WDC and VDC are competent and skilled enough to meet their objectives. This is demonstrated by their awareness of the community and its needs, as expressed by a male WDC member from Sokoto *“…what has helped me as the chairman of the WDC is my knowledge of the people living in the community*.*”* This in turn translates to trust and support for the leadership provided as community members can experience the capacity of the WDC and other structures. Additionally, WDC members make sacrifices to sustain the work. This includes providing funding from personal sources. *“Since I have my business*, *I support the WDC with my money as the leader of the WDC before calling other members of the WDC to give their financial support*.*”*

Capacity is also demonstrated by community committees’ successful implementation and perceived progress in enhancing their communities’ access to healthcare. There is also a sense of strong capacity to continue without support from health interventions such as the Breakthrough ACTION/Nigeria programme.

“*In reality*, *all the work I do*, *and everywhere I go*, *I am making great progress everywhere I go*, *because what is happening is that; it is not everywhere you go that people will refuse you*, *some places will even be happy that you are bringing this solution to them on the issue of health care*. *Truly I do not have problems often*. *Yes*, *VDC is strong to the extent that it can continue even without any support*.*”*

• **Sokoto, Male VDC Member**

### Community level factors affecting social cohesion

The community-level factors explored in this study were relationships and ties which comprise social capital and trust, process performance and goal attainment as well as self-reliance and collective self-efficacy.

#### Relationships and ties

*Social capital*. Social capital is demonstrated in the way community organizations leverage their committee and community members to obtain the resources necessary to achieve their goals of improving health behaviours and health outcomes. Examples of social capital utilization, as noted by the participants below, include the use of community resources like automobile owners providing transportation for medical emergencies.

“*We have contacts of a lot of car owners and motorcycle riders and if any emergency arises inside this community*, *I will call them immediately and they will come right away and take the person to the hospital for assistance*. *I believe that is an achievement*,*”*

• **Bauchi, Female WDC member**

Also, as noted by a female VDC member, community fundraising that accounts for different abilities to contribute is another example of community networks used to enable gains for the common good.

“*Very good*! *For instance*, *one would give five thousand and another who does not seem to have any financial strength will be taxed two thousand*, *some even one thousand*, *there are even others with five hundred*. *So that is how we were able to raise the funds*.*”*

• **Bauchi, Female VDC member**

A female CV underscored collaboration as necessary to leverage social capital.

“*We are all united*, *we have become like a broom tied together*. *Everyone has a role to play*. *When we hear any [health] challenge*, *we will look for this party and that other party…we CVs will not be enough*, *WDCs will not be enough…*. *[since we] joined hands*, *we have been having progress*.

• **Sokoto, Female CV**

*Trust*. Trust emerged as a fundamental element of cohesion among community members, and served as the cornerstone of all community endeavours that committee members implement.

Transparency and accountability help to build trust as evidenced by the prudent management of funds by WDC and VDC members. Participants also highlighted trust in health sensitization as a factor that has helped build trust and allowed for acceptance. Study participants emphasized that the endorsement and cooperation of influential traditional and community leaders, along with committee members, were vital for the successful reception of community sensitization and education efforts. Without their support and engagement, such initiatives would not be well received and could potentially encounter resistance.

Furthermore, LGA officials acknowledged that transparency plays a role in fostering trust within the community. It enables donors and other projects to view WDCs as reliable entities, thus facilitating SBC activities. committee and community members emphasized the importance of building trusting and accountable relationships. This opinion was shared by WDCs and VDCs.

“*Accountability and transparency are the key things that help me in my work*. *And I received a lot of support from members because they know I do not joke with their money*…. *The effective utilization of funds to address emerging health issues with the consent of other members for proper accountability and transparency*. *Every penny spent is duly accounted for*, *and this makes the people happy with the support and the assistance we render*.

• **Sokoto, Male VDC member**

“*…I know what I have done and what I am still doing for the progress of the community*, *I am loved*, *and everyone is happy I do not cheat*, *I have made an impact*, *and I can go to any extent for my community to be happy*.

• **Sokoto, Male WDC member**

The WDC membership elects its executive members for two-year terms based on their reliability, commitment to assist their community, and connection to the communities they serve. Responsibilities include facilitating the introduction and seamless integration of CVs for productive dialogues and visits. This crucial role ensures that the community warmly embraces the CVs and promotes their effective engagement.

“*Yes*, *we attend meetings with WDCs we also visit house to house with WDCs because there are times in which the community might look at us as too young but whenever we are led by our WDCs they give us a form of listening ears and prestige because WDCs are older people and are more respected within the community*.

• **Bauchi, Female CV**

#### Process performance and goal attainment

The majority of participants who were WDCs, VDCs, and CVs noted that they understood their roles and responsibilities, particularly when it came to health issues in the community. These roles include the improvement of access to health services, community engagement, fundraising, health facility maintenance, sensitization, and supportive supervision of CVs. The delimited roles facilitate performance and attainment of the goals conceived as part of their responsibilities. Participants stated that access to health services has been improved by community organizations through the provision of transportation and procurement of financial resources for out-of-pocket costs associated with medical services. WDCs also ensure that community member representation is geographically equitable to allow for more accurate accounting of health issues and more equitable distribution of health benefits.

“*For us*, *in the areas of health*, *we listen to the people’s problems*, *find a possible way of solving them sometimes by contributing money to make facilities comfortable for them*, *assist pregnant women with the car to have their babies*, *pay hospital bills*, *meet them in their houses to give advice and all*, *because a leader should be able to listen to the people*.*”*

• **Sokoto, Male WDC Member**

“*Also*, *for instance*, *if there is any advocacy or intervention that entails pregnant women or parents with children below the age of five years*, *automatically that is my role*… *I will then go ahead and mobilize women and ensure that the job is done*. *We choose members from every village to have equal representation from every village so issues peculiar to each village are tabled out one after the other to ensure no village is left out of our intervention*. *Thereby leading to equal benefits across villages*. *So also*, *if there is any material intervention*, *the commodity is equally shared amongst these representatives that came from the various villages*.*”*

• **Sokoto, Female WDC Member**

The WDCs are highly involved in educating the community members on health behaviours and the use of health services. Sensitization, as stated by one female WDC member, is viewed as a means by which health challenges may be addressed; she argues that *"The main job of WDCs is public awareness or enlightenment*, *regarding a number of health issues that can only be salvaged by educating the people*.*"* Furthermore, significant health subjects covered during sensitization include the usage of long-lasting insecticidal nets, child immunization, and antenatal care. Sensitization has yielded perceived results such as an increase in demand for antenatal care services and autonomy in health-related decision-making.

WDCs play a significant role in service delivery at health facilities by providing supportive supervision to community health volunteers. This obligation stems from the need to ensure the highest quality of service possible while also ensuring that community members are available and willing to receive it.

#### Collective self-efficacy and self-reliance

*Early successes in building self-efficacy*. Participants’ belief in their capacity became pronounced when discussing the community-based promotion of health information and behaviours.

All categories of study participants referred to the WDCs and VDCs as proactive agents driving positive change by improving health facilities and transportation conditions, thereby exhibiting a high level of capacity. Examples of their achievements include improving the infrastructure and availability of medical supplies at healthcare facilities, as well as mobilizing the community to utilize these facilities. Interviewees expressed confidence in the collective ability of these committees to sustain the work.

“*We will stand on our own*. *The VDC is already working with WDC to sustain the health programmes that BA is teaching us*. *This is done through the support of the district head*, *stakeholders*, *and other concerned citizens*. *We make decisions regarding our health facility and other health challenges confronting our community*. *We are on our way to attaining independence*.*”*

• **Sokoto, Male VDC member**

Examples of tangible measures made possible by WDCs in concert with the local government and communities which have reinforced their collective efficacy include outdated equipment, buying new beds, building benches for the waiting area, and providing fuel for vehicles used as emergency transportation for women in labor.

A few examples of concrete actions made possible by WDCs include replacing outdated equipment, buying new beds, building benches for the waiting area, and providing fuel for vehicles used as emergency transportation for women in labor.*“We succeeded; we brought electricity to our hospital*, *you see*, *we bought curtains and fixed them*, *we bought buckets to replace the old ones the government bought*, *we the members of WDC did that*.*”*

• **Bauchi, Female VDC member**

*Evidence of self-reliance*. Differences in perspectives arose when examining the community’s capacity to mobilize financial resources when necessary. While some participants assert that substantial progress can be achieved without relying on external funding, expressing confidence in advocating for private financial support as an opportunity, other individuals emphasize the limitations of their financial self-reliance. They perceive the withdrawal of external funding as a potential threat to the long-term sustainability of health programmes. The statement below is from a female WDC member who was confident in the collective capacity of the committee to mobilise financial resources.

“*We now know that we could raise funds within and outside our community by carrying out advocacy visits*, *letting our elites understand the yearnings and sufferings of the people in our community and seek support in areas of need…mostly pertaining to the health care services rendered within the community and explore how we can help alleviate the suffering of the less privileged members of our community*…*”*

• **Bauchi, Female WDC member**

Donations from committee members and financial support from well-known members of the community are the main sources of funding. Except for sensitization activities, other WDC members conveyed their perception of being unable to sustain the complete health programmes in the absence of external funding. This was reported by a male WDC member in Sokoto state: “*No*, *the community cannot sustain it [health programmes]*. *We do not have that strength*. *The job of sensitizing the people is the only thing we can do [without] outside funds because we have been doing it so we can continue*.*”*

Participants believed they can sustain activities that “*does not require any funds*” such as “*ccommunity meetings*”, “*getting women to come to the hospital*”, and “*any work that has to do with sweeping or repairs in the hospital*” as explained by two WDC members in Bauchi state.

#### Institutional level factors affecting social cohesion

The institutional-level factors explored in this study were conflict management and decision-making, human rights, and the environment as it includes its structure, norms, and values.

#### Conflict management and decision making

The factor of conflict management and decision-making is central to social cohesion at the institutional level. It is focused on limiting associated negative aspects including social disorganization or conflict and inequalities and exclusion. The WDCs and other community structures play a vital role in community conflict management and resolution, especially with regard to health issues. Their participation in dispute resolution is linked to their roles and goals as WDCs as well as the influence they wield in the community. Because of their standing in the community, WDCs are respected and trusted on health-related problems. According to several participants, WDCs serve as the first level of dispute resolution between community members in health-related matters. In addition, conflicts are resolved by promoting an understanding of the issue and seeking out individuals who could serve as mediators.

“*You see like polio injection*, *when they go*, *they do have some issues with some household*. *Someone may just decide and say they should not enter his house*. *We the WDC go and meet the household head*, *call him*, *and advise him on the issue*. *If he refuses*, *we take it further to the village head*. *The village head may go and meet him or send to call him*. *The village head will explain to him what is going on and*, *in the end*, *success is achieved*. *But if someone hardens like this and we try to force him it will result in hard feelings*. *Someone may say he will rather be dead*. *You see that is not what we are looking out for*. *What we want is to prevail and achieve the goal*.*”*

• **Bauchi, Male WDC Member**

In contrast, there was no consensus in reports of conflict within the committees nor conflict management approaches. Responses ranged from perceived absence of conflicts between members to conflict management technique bereft of a resolution pathway.

“*Honestly there is an agreement*, *we understand each other there is no difference*. *If anything comes up*, *everybody trusts and understands each other*. *There is nothing bigger than when you unite and work together*. *If we didn’t accept each other*, *we would not have been doing things together*. *There is no misunderstanding or disagreement between us*.*”*

• **Bauchi, Male WDC Member**

“*When we discover that there any misunderstandings or disagreements*, *if the problem is from our people*, *we confront the situation in an amicable manner*, *trying to know what happened*, *and settle the matter*.*”*

• **Bauchi, Female WDC**

#### Collective agency

On the institutional level of social cohesion, the effectiveness with which WDCs and VDCs perform job-related activities demonstrates their collective agency. Evidence of agency is the perceived competence to address relevant health challenges in the community. A male WDC in Bauchi highlighted the positive results of community engagement in this regard. He said, “*Our ability to interact with the community and relate to its members gives us confidence*. *Some of them come to us on their own to seek our guidance on what to do*.” Also, a Male VDC in Sokoto emphasized the involvement of committee members in addressing health issues in the community. He puts it this way,

*“I take every opportunity and do my best to address whatever issue that may arise in the community and I also involve other [VDC] members to help solve it through good relationship with people and the knowledge on health. That’s what helps me. Since we [VDC] have members, we sit and do what we ought to do.”*

On the other hand, WDCs and VDCs also provided evidence of limited agency due to gender roles and expectations. For instance, in the Northern region of the country, gender socialization has resulted in differing responsibilities for male and female members. The extent to which they can undertake their roles is limited. A male VDC member in Bauchi remarked that "*Female leaders are the ones tasked with going into women’s houses*, *which*… *is not in place for a guy to walk into even if it is to enlighten women*. *A man engages with other men to enlighten them*, *while the female leaders enter homes to enlighten the ladies*.*"* Another male VDC member from Sokoto elaborated on this further, saying, *“You know our tradition here does not permit a man to visit another man’s house*, *yet women may enter and see women*. *That is why the VDC has female members who visit homes to persuade other women to get ANC*, *family planning*, *and immunization*.*”*

**Environment (structures, norms, and values).** A key factor at the institutional level is the environment, (i.e., structures, norms, and values) as it relates to formal institutions and actors in the society that are responsible for its upkeep. This is evidenced by the existing hierarchy to address health-related issues in the community. Participants opined that community leaders, influential individuals, and community organizations are tasked with this responsibility. As noted by a female CV in Bauchi, those considered best qualified to address health concerns are characterised as “*Elderly*, *responsible and experienced in terms of their social and religious values*”. She cited the Traditional Ward Heads “*Mai-Unngwas*”, spouse, and a WDC member, as a befitting example of people that fall into this category.

“*Well*… *It is the Mai-Angwa and WDCs that are saddled with this responsibility*. *Whenever there is any issue that goes beyond the CV*, *and WDC the Mai-Angwa takes the final say because he is on top of the hierarchy of community decision making*.*”*

• **Bauchi, Male CV**

### Possible threats to sustainability of the community capacity strengthening approach

The sustainability of community efforts depends in part on social cohesion, but other elements and aspects may be threatened by challenges that the community organisation members may not be able to surmount, despite these positive social cohesion findings. These include challenges related to finances, logistics, and service delivery.

Our findings revealed that the resource mobilization strategies employed by WDCs primarily revolved around fundraising efforts within their membership or seeking contributions from influential community members. However, this approach may not be sustainable since not all WDC members possess the capacity or financial resources to make substantial donations or generate significant funds.

WDCs highlighted the burden of out-of-pocket expenses borne by community members as one of the foremost challenges in enhancing health outcomes. While WDCs request financial resources from the community for immediate use and to establish contingency funds for addressing future health concerns, it is crucial for WDCs to recruit and maintain members with financial resources to fund community projects. WDC members conveyed confidence in their capacity to rally community leadership to a certain extent. However, other WDC members expressed significant concerns when confronted with the need for additional funds for community projects.

“*There are tasks which we desire to carry out but because of the situation…*. *Looking at the financial strength of the community here in [name of village]*, *we desire to conduct an electrification project in this community*. *You see*, *this is something even though the community desires it*, *it is not capable of carrying out such a project for itself*.

• **Bauchi, Female WDC member**

This financial predicament can become even more complex when WDC members personally endure unfavourable socioeconomic conditions. In instances where committee members are unable to contribute their financial resources for community initiatives, they may be perceived as unwilling. Furthermore, WDC and VDC members frequently bear other obligations for generating income outside of their committee roles, which can sometimes pose conflicting priorities when engaging in collective action.

“*Taking women to access medical services in health facilities often conflicts with my role as a farmer*.

• **Sokoto, Male VDC member**

Moreover, community members frequently rejected the health messages that committee members and CVs came to offer because they frequently anticipated that committee members would give them a monetary inducement (such as cash, goods, or commodities) during home visits. These expectations encompassed items like mosquito nets, malaria medications, family planning commodities, or even direct cash assistance.

“*When mobilizing people for community dialogue…they sometimes resist saying they need to be compensated with money*.

• **Bauchi, Male CV**

Furthermore, insights from a Breakthrough ACTION/Nigeria staff revealed that the commitment, capability, and competence of LGA officials to offer effective supervision will significantly influence the long-term sustainability of the initiatives implemented under the Community Capacity Strengthening approach in support of community-level SBC, behavioural uptake, and overall improved health of the community.

“*The state primary health care and the primary health care department in the local government areas*, *how committed would they be in terms of ensuring the continued follow up*, *supportive supervision to these structures that we found are formed already*? *It is one thing to have them embedded in the law*, *it is even one thing to give them formed activities and it is another thing to ensure that they are doing the right thing*. *Who will follow them to ensure they are doing the right thing*?

• **Bauchi, Breakthrough ACTION/Nigeria staff**

Lastly, poor provider behaviour and the absence of free or subsidised health commodities could also threaten community capacity beyond what strong social cohesion can overcome.

“*…when our people [community members] visit the health care facilities*, *they should not be humiliated*. *Humiliation brings about the turn off to hospital visits*. *But when they are not humiliated*, *but attended to nicely whatever happens to them*, *they will go straight to the hospitals*. *When they get there and they are screamed at and what have you*, *they are not happy*. *So*, *they feel reluctant to go to the hospital*.

• **Bauchi, Male VDC member**

## Discussion

This study examined individual, community, and institutional elements of social cohesion within and among community structures that contribute to the health ecosystem in Nigeria, and their implications for community capacity strengthening and sustainability of SBC programmes. No observable differences in results were found between the two study states, which may be attributable to comparable cultural contexts.

At the individual level, our study highlights important findings on self-motivation and perceived level of capacity. This study revealed that committee members leverage regular meetings to discuss ideas on a variety of health issues. These meetings are vital for concurrence, and resolution of challenges and provides needed motivation for members. This finding is similar to what was found in a study among village health committees in northern India where monthly meetings enabled members to advocate for needed resources [45]. The meetings aid in relationship building and, in the process, help identify needed solutions. Recognition and legitimacy were operationalized through the formation of community organisations which in turn gives the members credibility. In addition, health committee elections allowed individuals to be a part of the formal establishment and thus share the organization’s identity and norms. This also leads to a level of self-motivation for assigned tasks. This finding differed from that of another study that explored social accountability in primary health care in three African countries where even though committee members were installed through an official event, it was mostly through selection and not election [46].

We found that even though gender-based recruitment of committee members was intended to be fair/equitable, women’s participation is mostly determined by availability and social norms. Similarly, as gleaned from the findings reported by Scott et al. (2017), social norms constrain women’s ability to participate actively in health committees because they prohibited them from speaking to men in their communities and discouraged travel outside the village [45]. These norms are rooted in gender inequality, limit the potential, and threaten the cohesion of the group. Also noted as a recruiting factor was education level. This could be connected to the benefits of education, which might make it easier for committees to access resources. Our study describes the self-efficacy and capacity of committee members to make improvements in health facilities and transportation conditions. This is consistent with studies that have documented interventions on community committees in Nigeria. A qualitative study that examined the influence of capacity building on WDCs’ ability to deliver high-quality healthcare services in Osun State, Nigeria found that training enabled WDCs to identify urgent healthcare needs and to mobilize local resources to address identified gaps. This led to the provision of hospital beds and basic equipment and the completion of facility building [28]. In addition, a study that examined an MNCH network of care intervention found that WDC participation was essential to ensuring the successful operation of the voluntary public transport scheme for health to serve remote areas through sustainable contributory schemes [38].

At the community level, our findings highlighted the importance of social capital to the function of committees. WDCs leveraged the influence and capabilities of networks of relationships in the community to achieve their goal. This is consistent with a study that highlighted the importance of leveraging community resources such as WDCs to encourage caregivers’ immunization uptake [47]. Similarly, a study that examined the maternal and neonatal networks of care approach showed that members of the WDCs were instrumental in sourcing funds for recurring costs for healthcare delivery through sustainable contributory schemes [38]. Leveraging social capital in their roles as committee members highlights the close relationship that exists within the group and between the committee and the community. This is also an asset that could serve as an important pillar of the sustainability of health interventions.

Trust was identified as an essential element of cohesion necessary for people to accept and implement SBC programming. A study that assessed community health workers’ experiences in integrated service delivery in India similarly revealed that community health workers’ intermediary position requires them to have trusting relationships with both their communities and actors in the health sector [48]. Poor support, accountability, and communication structures could influence these relationships and subsequently result in significant challenges in building trusting relationships with community members and actors in the health sector, leading to demotivation and tensions as a result of trying to accommodate conflicting interests and expectations [49, 50]. Furthermore, in a study that explored the role of local communities in the retention of health workers in rural Tanzania, trust was also underlined as a critical factor in the acceptance of services provided [51]. Lack of trust led to rejection and discrimination of health workers. Trust has also been identified as a positive influence on motivation [52], and it is therefore not surprising that trust was vital in the successful implementation of activities for committee members in our study.

At the institutional level, our findings demonstrated the conflict management skills of committee members. Social cohesion is said to reduce conflict and inequality [53]. This was the case in this study as it was shown that WDCs help in conflict management and resolution as a result of the influence they wield in the community and the trust between them and community members. Findings from this study showed that committee members understood their roles and responsibilities which allow for the performance and attainment of the goals. Additionally, the effectiveness with which members perform job-related activities was evidence of collective agency. In addition, shared norms and values were crucial for acceptance by the community. This also represents a level of conformity and identity of members with the committee as well as drive to meet their goals.

Our findings underline clear limits to the extent high social cohesion can contribute to community capacity to sustain health interventions. This study discovered that significant limitations with funding, logistics, and service delivery were potential hurdles to the sustainability of the health intervention’s benefits. Funding especially is a significant challenge given the poverty profile of the study states. For example, poverty incidence is as high as 91% and 74% in Sokoto and Bauchi states respectively [54]. Consequently, it could be challenging to sustain health programmes from out-of-pocket donations. Committee members may need to embrace more flexible fundraising strategies that are not limited to the group and a few community members, since this may not bode well for sustainability of efforts. It is hardly surprising that supply does not meet the demand for healthcare services. This might indicate the effectiveness of the WDCs and other community institutions in creating demand for health services. It also suggests that the efforts put into bolstering healthcare supply do not complement those invested in healthcare demand generation. It might imply investment of resources to aid healthcare supply to guarantee that there is no reversal in improved health-seeking behaviour and attitudes.

### Study limitations

Data was not collected from comparison sites to study wards that did not receive the capacity strengthening intervention and understand whether and how social cohesion manifests itself. Additionally, the study team was unable to interview a key female WDC chairperson due to site inaccessibility and unresponsive ethics committee. This missed opportunity could have offered insights into female leadership within the WDC structure.

Lastly, qualitative data has inherent limitations in generalizing findings beyond the study participants and geography. Nevertheless, our study findings retain their significance and hold value for the implementation of SBC programmes. Our findings present a comprehensive depiction of different elements of social cohesion in the context of a Community Capacity Strengthening intervention, including reflections on their potential contribution to sustainability of health improvements.

## Conclusion

Our study findings demonstrate a high level of social cohesion among community organisation members which has advanced the implementation of integrated health programmes in the community. In addition, we found that the contribution of social cohesion to community capacity is limited by gaps related to funding, logistics and service delivery. While cohesive community health committees present a good opportunity for the implementation of health programmes, there is a need for further investment in the capacity of committee members and the health facilities they support. Beyond efforts of donors and initiatives like Breakthrough ACTION/Nigeria, the government at national and sub-national levels must assume responsibility and make the necessary investments, while other investment opportunities from the private sector could be explored to ensure the sustainability of community efforts to improve their health and well-being outcomes.

## Supporting information

S1 Appendix Inclusivity in global research questionnaire. (DOCX)

## Abbreviations

ANC: Antenatal care
CV: Community volunteer
IDI: In-depth interviews
KII: Key informant interviews
LGA: Local government area
MNCH+N: Maternal, neonatal, child health and nutrition
VDC: Village development committee
WDC: Ward development committee
